# Motivational Message Framing Effects on Physical Activity Dynamics in a Digital Messaging Intervention: Secondary Analysis

**DOI:** 10.2196/41414

**Published:** 2023-04-21

**Authors:** Alexandra M Lee, Sahar Hojjatinia, Jimikaye B Courtney, Deborah Brunke-Reese, Sarah Hojjatinia, Constantino M Lagoa, David E Conroy

**Affiliations:** 1 The Pennsylvania State University University Park, PA United States

**Keywords:** physical activity, exercise, fitness, Fitbit, tracking, patient-specific modeling, dynamical model, patient specific, fitness tracker, psychological theory, messaging, motivation, behavior change

## Abstract

**Background:**

Digital smartphone messaging can be used to promote physical activity to large populations with limited cost. It is not clear which psychological constructs should be targeted by digital messages to promote physical activity. This gap presents a challenge for developing optimal content for digital messaging interventions.

**Objective:**

The aim of this study is to compare affectively framed and social cognitively framed messages on subsequent changes in physical activity using dynamical modeling techniques.

**Methods:**

We conducted a secondary analysis of data collected from a digital messaging intervention in insufficiently active young adults (18-29 years) recruited between April 2019 and July 2020 who wore a Fitbit smartwatch for 6 months. Participants received 0 to 6 messages at random per day across the intervention period. Messages were drawn from 3 content libraries: affectively framed, social cognitively framed, or inspirational quotes. Person-specific dynamical models were identified, and model features of impulse response and cumulative step response were extracted for comparison. Two-way repeated-measures ANOVAs evaluated the main effects and interaction of message type and day type on model features. This early-phase work with novel dynamic features may have been underpowered to detect differences between message types so results were interpreted descriptively.

**Results:**

Messages (n=20,689) were paired with valid physical activity monitoring data from 45 participants for analysis. Received messages were distributed as 40% affective (8299/20,689 messages), 39% social-cognitive (8187/20,689 messages), and 20% inspirational quotes (4219/20,689 messages). There were no statistically significant main effects for message type when evaluating the steady state of step responses. Participants demonstrated heterogeneity in intervention response: some had their strongest responses to affectively framed messages, some had their strongest responses to social cognitively framed messages, and some had their strongest responses to the inspirational quote messages.

**Conclusions:**

No single type of digital message content universally promotes physical activity. Future work should evaluate the effects of multiple message types so that content can be continuously tuned based on person-specific responses to each message type.

## Introduction

### Background

Approximately half of adults in the United States do not attain recommended levels of health-enhancing aerobic physical activity [1]. Given the widespread lack of physical activity in the US population, innovative methods with high potential reach are needed to improve public health. One inexpensive mode for delivering physical activity interventions at scale involves digital smartphone messaging. Determining which validated targets to engage with different messages is a persistent challenge for content development and intervention delivery. Comparing person-specific behavioral responses to different types of intervention content can inform both intervention and theory development by extending target validation research from group-level to person-level analyses. However, limited research has compared the proximal effects of different message types on physical activity behavior after message receipt. This study compared the effects of different motivational message types on physical activity behavior after message receipt.

### Digital Messaging Interventions

Digital messaging interventions have the potential to reach large portions of the young adult population because 97% of young adults currently own a smartphone [2]. Smartphones are highly accepted by participants in physical activity promotion research, and intervention delivery via digital messages is a low-cost method for instigating behavior change [3,4]. In a meta-analysis, digital messaging interventions significantly increased device-measured steps per day (*d*=0.38) [3]. This effect exceeded the 90th percentile for physical activity interventions according to recent benchmarks for digital intervention effects on physical activity [5]. Despite the acceptability, feasibility, and effectiveness of using digital messaging interventions, little is known about the most effective types of messages for increasing physical activity.

A scoping review of physical activity messaging interventions proposed that messages should be framed positively and highlight beneficial short-term outcomes related to social and mental health, be tailored to the recipient, and use psychological theory and social marketing principles [6]. This framework does not specify which psychological theories should guide content development or which behavior change techniques should be incorporated. Many physical activity interventions are grounded in social-cognitive theories, such as the Theory of Planned Behavior and Health Action Process Approach [7]. These theories posit that attitudes (ie, positive outcome expectations), subjective norms, and perceptions of behavioral control are precursors for intention formation and that planning processes mediate the translation of intentions into physical activity behavior [8,9]. Emerging work also suggests that linking physical activity with desirable affective experiences can activate affective processes that motivate physical activity [10,11]. Consistent across these approaches is the idea that persuasive messaging can be used to frame the benefits of physical activity and influence decisions to be active.

### Attitude Change Validated as a Target for Physical Activity Promotion

Attitudes represent evaluative beliefs about an activity’s consequences and can be either instrumental (focusing on social or tangible costs or benefits) or affective (focused on positive or negative affective experiences) [9]. Changing attitudes has a medium-sized effect on intention strength and a small effect on behavioral outcomes [12]. Comparisons of affective attitudes and instrumental attitudes toward physical activity have revealed that affective attitudes are more strongly associated with physical activity intentions than instrumental attitudes [13]. Evaluated with more granularity, proximal within-person affective attitudes have been shown to predict exercise likelihood each day, more than distal affective attitudes or distal instrumental attitudes [14]. Proximal and distal refer to temporal proximity between the proposed cue or attitude and the intentions for, or actioning of, the behavior of interest. These findings suggest that affectively framed messages may be more effective than instrumentally framed messages for strengthening proximal physical activity intentions and subsequent behavior.

Three experimental studies have evaluated the effects of affective or instrumental benefit messages on physical activity behavior directly. Two studies favored affectively framed messages and one study showed no difference between affectively and instrumentally framed messages [15-17]. Each of these studies focused on self-reported physical activity outcomes 1 to 3 weeks after message delivery. The proximal effects of affectively and instrumentally framed messages on physical activity in the minutes and hours after message delivery are not known and were the focus of this study. To identify the dynamics between message receipt and response and understand potential person-specific responses to message types, a dynamical modeling approach is needed.

### Addressing Treatment Heterogeneity With Person-Specific Dynamic Modeling of Physical Activity

Device-based measures of physical activity can provide minute-level data of movement throughout the day. Step counts represent a valid measure of total physical activity volume accumulated throughout the day that is associated with cardiometabolic risk reduction, easy to measure, and widely accessible in consumer devices [18]. Approximately 70% of variability in physical activity occurs within people over time so this study focused on proximal changes in a person’s physical activity after message delivery [19-21].

Prior work from our group applied system identification methods to develop dynamic models that described physical activity over time and the proximal effects of digital messages on that behavior [22-24]. Behavioral responses to digital messages varied as a function of message content from weekends to weekdays and from person to person. In that work, message content was differentiated by the desired behavioral outcome (eg, move more vs sit less). Messages in the move more and sit less content libraries provided prompts or cues to form intentions to increase physical activity and also systematically varied in whether they were framed in terms of affective outcomes of physical activity or a combination of instrumental outcomes of physical activity and social-cognitive strategies for behavior change like goal setting, planning, identifying barriers, and engaging social support. We supplemented messages targeting instrumental outcomes with social-cognitive principles based on prior evidence that affective attitudes are more strongly associated with physical activity intentions than instrumental attitudes [13,15]. For simplicity, these 2 message types are described as affective and social-cognitive hereafter. This study examined whether message framing impacts proximal changes in physical activity.

### This Study

This study was a secondary analysis of data collected from a digital messaging intervention in insufficiently active young adults (18-29 years) who wore a Fitbit for 6 months during their waking hours and received 0 to 6 messages per day at random times. Methods from control systems engineering were used to identify person-specific models of physical activity and message effects on subsequent physical activity. The details of this methodology have been reported previously [22,23,25]. Briefly, we generated person-specific dynamical models of physical activity for each participant and analyzed the impulse response and cumulative step response curves for each message type (ie, affective, social-cognitive, and inspirational quotes). Model coefficients were used to simulate impulse and cumulative step responses to describe proximal behavior changes as a function of message type (separately for weekdays and weekends). This analysis was exploratory with the focus of identifying intervention responses across message types and day types. Person-specific modeling helps us better understand whether one message type outperforms the others across the sample or whether future work should consider personalizing message types for optimal performance from each participant.

## Methods

### Participants

From April 2019 to January 2020, we recruited emerging and young adults using ﬂiers and web-based advertisements. Eligible participants were 18 to 29 years of age, ambulatory, free of functional activity limitations, free of visual impairment that would interfere with smartphone use, had verbal and written ﬂuency in English, and were capable of giving informed consent. Participants also needed to own a smartphone using the iOS (version 10 or later) or Android (version 7 or later) operating system. Participants were excluded if self-reported physical activity levels were greater than 90 minutes of moderate- or greater-intensity physical activity per week; if they were unable to be physically active or had medical contraindications for physical activity; or if they were pregnant (or planning to become pregnant during the study period) or had a prior diagnosis of cancer, cardiovascular disease, diabetes, or metabolic disorder. Participants completed a telephone screening with research staff followed by a 1-week ambulatory monitoring period wearing an Actigraph wGT3X-BT activity monitor. Participants were excluded if the device recorded the equivalent of or more than 150 total minutes of moderate- or greater-intensity physical activity with a minimum of 5 days with at least 10 hours of monitor wear time during the 1-week monitoring period. The measures, protocol, and data preprocessing methods have been reported in detail elsewhere [22]. This analysis only includes participants who finished all data collection procedures before the onset of the COVID-19 pandemic.

### Protocol and Measures Overview

At the first laboratory visit (day 1), participants completed written informed consent for the screening run-in and received an Actigraph wGT3X-BT activity monitor to wear on the waist for the next week during waking hours. Participants were provided a paper wear log to record times of wear and nonwear. During the ﬁrst laboratory visit, participants self-reported demographic characteristics including age, ethnicity, race, sex, educational attainment, employment status, and occupation. Research staff measured height and weight in duplicate using a wall-mounted stadiometer and a digital scale upon removal of the participant’s shoes.

At the second laboratory visit (day 9), the researcher collected the activity monitor and wear log, downloaded data, reviewed nonwear classiﬁcations with the “Troiano 2007” algorithm in the ActiLife version 6.13.4 software, and determined eligibility with established cut points used to classify minutes as moderate (1952-5724 counts per minute) and vigorous (>5724 counts per minute) physical activity [26,27]. Eligible participants were briefed on the intervention phase of the study, and written informed consent was obtained for those who wished to enroll. Research staff then assisted the participants with installing the custom-designed study mobile app (Random AIM app) and Fitbit mobile apps on their smartphone and provided the participants with a Fitbit Versa/Versa Lite smartwatch. The participants were instructed to wear the Fitbit on their nondominant wrist to track step counts during the 6-month intervention period. This device recorded minute-level step counts and heart rate (in 5-minute moving averages). Participants provided an availability window of at least 10 hours for receiving messages on weekdays and weekends.

For the next 6 months, the Random AIM app delivered 0 to 6 messages/d as notiﬁcations via the operating system. The number, timing, and content of messages were determined at random each night with the constraints that no message could be delivered within 15 minutes of the previous message or outside the messaging window for that day. Messages were drawn randomly from 3 content libraries: affectively framed (54 messages), social cognitively framed (54 messages), and inspirational quotes (27 messages). The affective and social-cognitive libraries were both evenly split between messages focused on moving more and sitting less. Affectively framed messages additionally presented information about emotional consequences of engaging in those behaviors. Social-cognitively framed messages incorporated information about health consequences of engaging in those behaviors and included a prompt for goal setting, action planning, social support, or problem solving. The third message library, inspirational quotes, did not reference physical activity or changing movement patterns and did not prompt the use of any self-regulatory strategies aimed at promoting movement. See Textbox 1 for message examples. Half of the messages in each library were accompanied by an image corresponding to message content (ie, physical activities, standing activities, and natural landscapes). Research staff contacted participants via telephone or email anytime they observed 3 consecutive days without Fitbit heart rate data (suggesting device nonwear) or 3 days without acknowledging Random AIM messages.

Participants completed a ﬁnal laboratory visit (day 190) after the 6-month intervention period to remove the study apps.

**Examples of message types.**

**Affective**

- “No matter how slow you go, you are lapping everyone on the couch! Feel accomplished starting with just a few steps today”
- “Feeling down? Stand up and your mood will follow #SitLess”

**Social-cognitive**

- “Good news—every minute of exercise enhances health. Can't fit in 30 min today? Get what you can #MoveMore”
- “Need a cue to interrupt long periods of sitting? Try to stand every time you check your social media #SocialStanding”

**Inspirational quotes**

- “It always seems impossible until it’s done.” (Nelson Mandela)
- “Never let the fear of striking out keep you from playing the game.” (Babe Ruth)

### Data Analysis

#### Preprocessing

Three data tables were merged using timestamps to model physical activity dynamics following messages: person-level availability for messages, minute-level physical activity, and minute-level heart rate. Physical activity and heart rate data were included for the period from 2 hours before the messaging availability window started to 2 hours after it ended to ensure sufﬁcient activity data and to take into account messages received at the beginning and end of the window. Activity data were separated for weekdays and weekends and classiﬁed as missing if zero steps were recorded and heart rate data were not available for a minute. If the missing minutes were smaller than or equal to 3, step counts for those minutes were interpolated using linear interpolation. But minutes with missing heart rate and zero step counts of an interval of more than 3 minutes were not included in the model. Messages scheduled and sent from the server that were not received and displayed on a participant’s device were also not included in estimating the models. The available and valid minute-level physical activity data were aggregated into sums for each 15-minute epoch. Days were treated as independent; therefore, message effects on physical activity were not modeled across days.

#### System Identification

The Python programming language was used to implement the system identiﬁcation algorithms developed to identify the models [28]. Building on prior work, physical activity was modeled as a switched system with separate models to reﬂect the different amount and patterns of physical activity on weekdays and weekends [23,29]. The linear regression model with multiple variables and noise is of the form



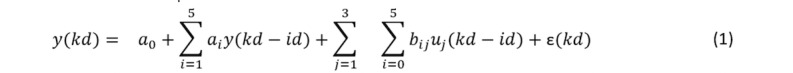



where *y*(*kd*) is the system output at time *kd*, which is the predicted step counts at time *kd*, *u_j_*(*kd*–*id*) are the inputs for the 3 message types (affective, social-cognitive, and inspirational quotes) at time *kd*–*id* (0: message not sent and 1: message sent), *d* is the sampling time (15 minutes), *ε*(*kd*) is noise at time *kd*, and *a_0_*, *a_i_*, and *b_ij_* are the unknown coefficients of the model. The trade-off between model complexity and size of the model error led to the model order of 5, which means that, in addition to the present 15-minute epoch of input data, the last 5 epochs or 75 minutes of both input and output data were used in predicting the output (step count) at the present epoch. The system coefficients are identified using the least-squares method by minimizing the square root of residuals as



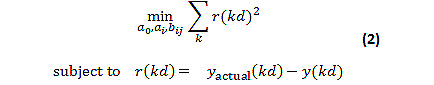



where *y_actual_*(*kd*) is the actual step count recorded by the activity monitor or linearly interpolated if the missing minutes were less than or equal to 3 and *y*(*kd*) is the predicted step counts at time *kd*. Models from both stages of analyses (weekdays and weekends) were used to simulate responses to each message type. Impulse responses represent expected step count changes during each 15-minute epoch after receipt of each message type (compared with expected step counts had a message not been received). Cumulative step responses represent the total expected effect of each type of individual message. Error bounds were estimated for each response curve to indicate whether effects exceeded the threshold of noise in the model.

Seven features were extracted from the simulated impulse and cumulative step response curves [23]. Each feature was extracted separately for weekends and weekdays. These features include initial delay, peak magnitude, peak delay, steady state, rise time, settling time, and effective time. *Initial delay*, *peak magnitude*, and *peak delay* were extracted from the simulated impulse response curve. Initial delay is the latency to initiate a momentary message effect (in other words, the time delay between receiving a message and showing a response via a change in step counts), peak magnitude is the magnitude of peak momentary message effects, and peak delay is the latency to peak momentary message effects. The *steady-state* value is the ultimate amount of the cumulative step response. *Rise time* is the time that it takes for the cumulative step response to advance from 10% to 90% of the steady state. *Settling time* describes the time that the step response enters a boundary around the steady state with the upper and lower bounds being 95% and 105% of the steady state, respectively. *Effective time* is the duration that the system response is above the noise level and has a detectable effect (response is outside the error bounds).

### Statistical Analysis

Descriptive statistics for mean, SD, and range were calculated for each model feature segmented out by message and day type. We conducted a series of 2-way repeated-measures ANOVAs with within-person factors for message and day type and each model feature as an outcome to understand the main effects of message and day type and their potential interaction. Two model features, effective time and peak delay, did not meet the normality assumption; thus, we conducted the Friedman test for these 2 features looking at the main effects of message type on each model feature within data sets for weekdays and weekends. Effect sizes were calculated as η^2^ for the 2-way repeated-measures ANOVA and Kendall *W* for the Friedman test. For main effects that were found to be statistically significant, multiple pairwise *t* tests (Wilcoxon signed-rank for nonparametric) with a Bonferroni correction were calculated to identify which groups significantly differed. Statistical analyses were completed in R Statistical Software (version 4.2.0; R Core Team) [30].

### Ethics Approval

All procedures were approved by the institutional review board at The Pennsylvania State University (Study#00009455). All participants provided informed consent to participate in the study and for their data to be used in study analyses. All participants were assigned a study ID number to link their data and remove personally identifiable data from the analytical data set. Participants were compensated up to US $295 for completing all study activities.

## Results

A complete participant flow diagram is available from the primary paper [22]. The average age of participants (n=45) was 24.4 (SD 3.1) years, and the sample was 67% female, 64% White, 22% Black, 9% Asian, and 4% mixed race. Overall, 20,689 messages were paired with valid physical activity data for analysis, and received messages were distributed as 40% affective (8299/20,689 messages), 39% social-cognitive (8187/20,689 messages), and 20% quotes (4219/20,689 messages).

Table 1 shows the means, SDs, and ranges for 6 model features extracted from person-specific dynamic models separated by day and message type. Initial delay was uniformly zero for this data set; thus, we focus on reporting results based on the other 6 features. Descriptively, the ranges present in this table show that there is significant heterogeneity in participants’ behavioral responses to messages, especially on weekends when more extreme behavior change was observed for all message types.

Table 2 presents the results from a series of 2-way repeated-measures ANOVAs with message and day type as within-person factors and each model feature as the outcome of interest. The data were not normally distributed for effective time and peak delay; thus, the Friedman test was implemented to evaluate the relationship between message type and these 2 features, respectively. Table 2 shows that there were no significant interactions between message type and day type for any of the model features. Significant main effects were observed for day type with rise time, settling time, and peak magnitude, meaning that the time it took for a message to go from low to high effect, the time that the effect settles around and close to steady state, and the magnitude of maximum momentary message effects were significantly associated with day type. Multiple pairwise comparisons revealed that each of these model features was significantly larger on weekends compared with weekdays (all *P*-adjusted *P*<.01).

Significant main effects for message type were observed with peak magnitude, meaning that the magnitude of maximum momentary message effects differed significantly by message type. Multiple pairwise comparisons of these significant main effects revealed that affective (*P*<.001) and social-cognitive messages (*P*=.02) had a significantly larger peak magnitude than inspirational quotes messages.

**Figure 1:**
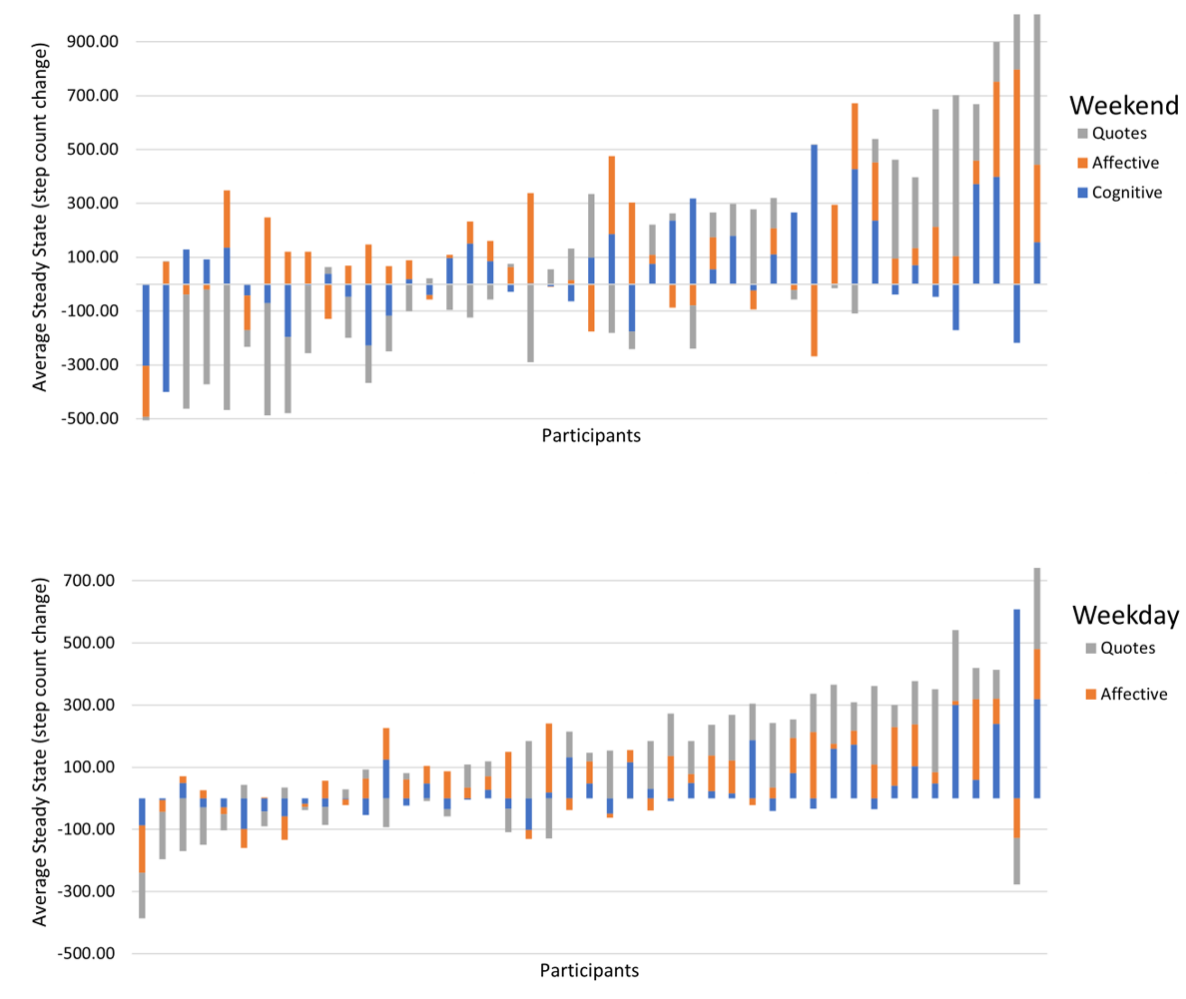
Participant steady-state averages for affectively framed, social cognitively framed, and inspirational quotes messages on weekends and weekdays.

**Figure 2:**
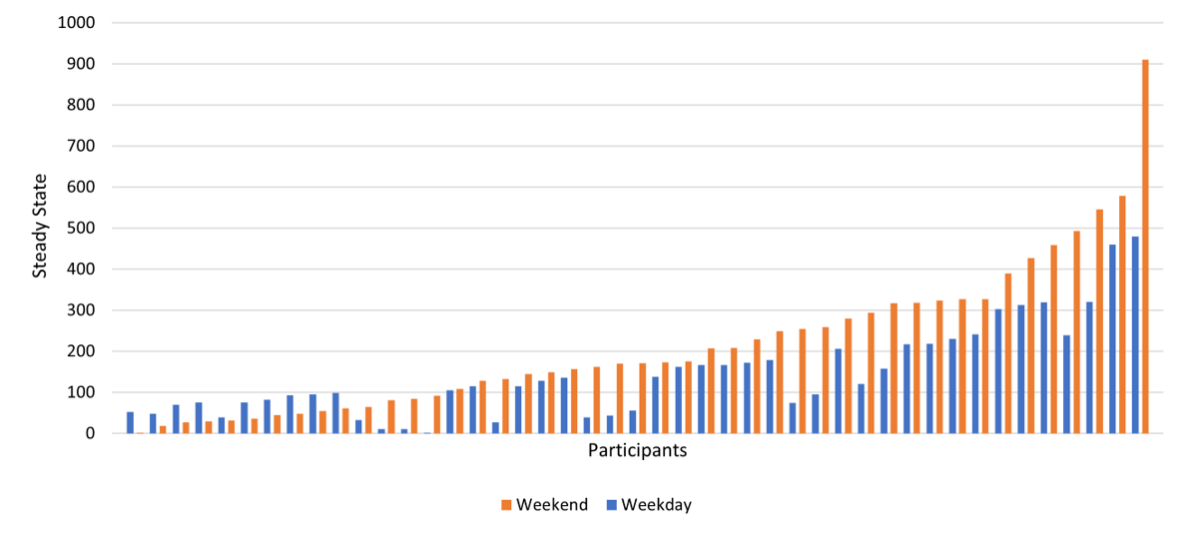
Participant-level differences between message types with the maximal and minimal average steady-state response on weekends and weekdays.

Figure 1 shows the average steady state for each message type per participant separated by weekends and weekdays arranged by order of magnitude. Steady-state responses reflect the overall magnitude of message effects, so we believe that they are more informative to focus on compared with peak magnitude, which only represents the expected behavior change during a single 15-minute epoch. This figure presents two key descriptive findings from this analysis: (1) there is heterogeneity in the range of average responses and responses by message type between participants, and (2) looking within each person, average participant responses to intervention messages differed by message type. Regarding the first, the minimal average responses are below zero and the maximal average responses are close to 800, and the colors show that across participants, the order varies for which messages produce the greatest and the least response. Regarding the second, each individual bar shows variation within each participant about their average message type preferences. Just over one-third of participants differed by more than 250 steps per message between the different intervention message types delivered to them (shown in Figure 2). Thus, on an individual level, if a participant received the 3 messages of the optimal type, they would be expected to increase daily physical activity 750 steps more than if they received the same dose of their least optimal message type.

**Table 1:** Description of model features by day and message type.

Feature		Affective				Social cognitive				Inspirational quotes		
		Mean	SD	Range	Mean		SD	Range	Mean		SD	Range
**Steady state**												
	Weekday	48.7	87.7	−153.0 to 259.8	48.8		129.3	−101.9 to 608.7	51.9		117.7	−169.5 to 266.4
	Weekend	90.8	181.1	−268.0 to 796.6	49.6		191.5	−400.1 to 516.8	12.3		265.0	−466.9 to 753.9
**Rise time**												
	Weekday	76.3	47.1	0 to 165	79.0		49.4	0 to 240	71.7		39.3	0 to 165
	Weekend	89.0	62.9	0 to 210	88.7		62.2	0 to 270	96.0		65.4	0 to 270
**Settling time**												
	Weekday	134.7	47.9	60 to 240	151.3		56.0	60 to 330	139.0		46.7	60 to 270
	Weekend	231.7	60.6	60 to 345	182.7		100.2	75 to 480	179.0		71.9	45 to 375
**Effective time**												
	Weekday	32.0	213.4	15 to 600	158.7		222.0	15 to 600	221.3		259.4	15 to 600
	Weekend	50.5	266.9	15 to 600	234.0		262.7	15 to 600	252.7		280.1	15 to 600
**Peak magnitude**												
	Weekday	32.0	14.8	3.3 to 67.6	37.7		26.4	10.1 to 156.4	50.8		25.5	16.5 to 120.9
	Weekend	50.5	24.7	14.5 to 121.9	55.3		28.7	10.6 to 126.6	63.8		39.0	8.2 to 196.9
**Peak delay**												
	Weekday	30.0	21.9	0 to 60	26.3		23.5	0 to 60	29.3		22.4	0 to 60
	Weekend	28.3	24.2	0 to 60	23.7		22.0	0 to 60	27.7		23.1	0 to 60

**Table 2:** Repeated-measures ANOVA results for message and day type main effects and interactions.

Feature and Effect			*F* or chi-square test statistic (*df*)		*P* value		η^2^ or Kendall *W*
**Steady state**							
	Message	1.074 (2, 88)		.35		0.008	
	Day	0.003 (1, 44)		.96		0.00001	
	Interaction	1.376 (2, 88)		.26		0.009	
**Rise time**							
	Message	0.021 (2, 88)		.98		0.0001	
	Day	5.484 (1, 44)		.02		0.020	
	interaction	0.815 (2, 88)		.45		0.003	
**Settling time**							
	Message	0.802 (1.7, 76.0)		.50		0.003	
	Day	18.868 (1, 44)		<.001		0.075	
	Interaction	0.307 (1.8, 76.9)		.71		0.001	
**Effective time^a^**							
	Message on weekdays	3.53 (2)		.17		0.039	
	Message on weekends	0.504 (2)		.78		0.006	
**Peak magnitude**							
	Message	10.987 (1.7, 76.3)		<.001		0.057	
	Day	34.429 (1, 44)		<.001		0.084	
	Interaction	0.423 (1.8, 77.1)		.63		0.002	
**Peak delay^a^**							
	Message on weekdays	0.824 (2)		.66		0.009	
	Message on weekends	0.658 (2)		.72		0.007	

^a^Required nonparametric tests.

## Discussion

### Principal Findings

We conducted a 6-month intervention to promote increases in step counts in insufficiently active young adults via digital messages. This secondary, exploratory analysis compared intervention responses to affectively framed, social cognitively framed, and inspirational quotes messages to identify if one message type elicited a consistently greater intervention response after the delivery of one message. Using system identification, we generated person-specific dynamical models of physical activity and found that step responses did not statistically significantly differ by message type, but the speed and momentary magnitude of intervention and step response was greater on weekends compared with weekdays for all message types. We also observed significant participant heterogeneity such that some participants achieved their highest steady state from affective messages (weekdays: 35.6%, weekends: 37.8%), some from social-cognitive messages (weekdays: 26.7%, weekends: 35.6%), and some from inspirational quotes (weekdays: 35.6%, weekends: 26.7%). Thus, this exploratory analysis suggests that personalizing message types for participants in an intervention may be a worthy endeavor for generating greater step responses over time.

Prior research has yielded mixed results regarding whether affectively or instrumentally framed messages were more effective at promoting physical activity [15,17]. Our results suggest that message effectiveness may be person-specific given the large ranges in steady state and the lack of statistically significant main effects by message type. The cognitive-affective system theory of personality may help provide further explanation for why we see varied, person-specific responses to intervention messages [31]. This theory proposes that networks of cognitive and affective processing units are activated when an individual processes a situational feature, like an intervention message [31]. The specific nature of cognitive and affective processing units activated by messages account for individual differences in behavior change following a message [31]. Within each person, this network of cognitive-affective processing units produces predictable patterns of behavior across time in response to specific situations that activate a network [31]. Thus, applying this theory to our findings suggests that participants each have different systems of cognitive and affective processing units that result in idiosyncratic physical activity behavioral responses to different message types. Identifying the patterns of these participant-specific behavioral responses over time can inform the selection and timing of message content.

The favorable responses of some participants to the inspirational quotes were unexpected given that these messages were purposefully not based in behavior change techniques or theories of behavior change and were intended to serve as a simple comparator. It may be that the inspirational quotes generated an affective response that stimulated playful, exploratory behavior (cf. broaden and build theory) or intentions to be active; however, we are unable to discern the mechanistic process from this analysis [32]. One observation that aligned well with past literature was that step responses differed on weekends and weekdays [23]. Message framing effects may depend in part on the social context in which they are received. Given the reduced magnitude of step responses to all message types on the weekdays, this difference could reflect an environmental constraint, such as work or school, that prevented action after the delivery of an intervention message. Personalization approaches that identify optimal times for message delivery may be especially valuable on weekdays.

This work echoes our prior work that showed differences in step count responses by day type and significant participant heterogeneity in response to message type; however, our prior work focused on move more and sit less messages as opposed to affectively framed and social cognitively framed messages [22]. The median effect of digital physical activity interventions in adults is 943 steps per day [5]; thus, if a future intervention included multiple messages per day, knowledge of optimal participant response could become meaningful because approximately one-third of this sample showed a minimum of a 250-step difference between message types. This heterogeneity between participants indicates that future interventions can benefit from methods that can both explore the effects of multiple message types on physical activity and exploit the most effective message types for an individual once identified. Given that messages have proximal effects on behavior in the minutes and hours after message delivery, the use of wearable devices for measuring physical activity behavior provides a rich source of information about behavioral dynamics. Harnessing this technology, system identification and dynamical modeling can inform future work that continuously tunes interventions based on participants’ responses over time [25].

This study used innovative person-specific dynamic modeling of intensive longitudinal data collected from a small sample of participants. This secondary analysis shifted from a within-person intervention designed to expose participants to a variety of message types repeatedly over 6 months to a paired-samples comparison of message types. Based on the design, the analyses are likely underpowered for detecting small- to medium-sized differences in these novel features of response dynamics. Other psychosocial and environmental factors could be influencing step counts that we are unable to account for in our models. However, the random aspect of our message delivery and type should mitigate the impact of potential confounding factors on our results. Conclusions may not generalize to other age groups given that messages were written for a young adult audience.

### Conclusions

Inactive young adults may benefit from digital messaging interventions to promote increases in step counts. In this sample, there was not a consistent difference in step responses to affectively framed versus social cognitively framed messages. Instead, participants demonstrated heterogeneity in which message type elicited their highest average step response, with some showing more preference than others. Future work should consider incorporating multiple message types so that content can be continuously tuned to the individuals who respond more favorably to the specific types of messages.

## Data Availability

The data sets generated and analyzed during this study are available from the corresponding author on reasonable request.
